# Otomastoiditis Caused by *Mycobacterium abscessus*, the Netherlands

**DOI:** 10.3201/eid1601.090473

**Published:** 2010-01

**Authors:** Jakko van Ingen, Frank Looijmans, Piet Mirck, Richard Dekhuijzen, Martin Boeree, Dick van Soolingen

**Affiliations:** Radboud University Nijmegen Medical Centre, Nijmegen, the Netherlands (J. van Ingen, F. Looijmans, R. Dekhuijzen, M. Boeree); National Institute for Public Health and the Environment, Bilthoven, the Netherlands (J. van Ingen, D. van Soolingen); Academic Medical Centre, Amsterdam, the Netherlands (P. Mirck)

**Keywords:** bacteria, atypical mycobacteria, atypical Mycobacterium infections, Mycobacterium abscessus, Mycobacterium avium complex, tuberculosis and other mycobacteria, mastoiditis, otitis media, the Netherlands, letter

**To the Editor:** Nontuberculous mycobacteria (NTM) are increasingly recognized as human pathogens (*1*). Otomastoiditis is a rare extrapulmonary NTM disease type first described in 1976; *Mycobacterium chelonae–M. abscessus* group bacteria, which are rapidly growing NTM, are the most frequent causative agents and the disease mostly affects children (*1**–**3*). In the Netherlands, *M. chelonae–M. abscessus* group isolates have been reported from the otologic samples of an average of 2 patients annually since 2006, as compared to 6 patients in the preceding 10 years. This emergence is not a likely result of improved laboratory facilities or awareness in clinicians because liquid culture and molecular identification techniques predate the rise in notification and Dutch guidelines advise against performing cultures for chronic otorrhea.

We identified 10 patients from the national reference laboratory database with otologic samples yielding *M. chelonae–M. abscessus* group bacteria during January 1995–June 2007. We resubjected these isolates to molecular identification by *rpoB* gene sequencing (*4*) and performed a medical file review.

The *rpoB* gene sequencing showed that *M. abscessus* was the causative agent in all 10 cases; *M. abscessus* seems to have a predilection to cause otomastoiditis. Closely related *M. bolletii* and *M. massiliense* (*4*) were not found. Early reports identified *M. fortuitum* or *M. chelonae* as causative agents, which may be because the taxonomy of the rapidly growing NTM has long been debated (*2**,**4**–**7*); many of these agents may have, in fact, been *M. abscessus*. All primary isolates were found susceptible to clarithromycin and resistant to fluoroquinolones and aminoglycosides in our agar dilution method (*8*), which is not the recommended method for rapidly growing NTM (*1*). Two patients acquired clarithromycin resistance during treatment.

Clinical data are summarized in the Appendix Table and match those of previous studies (*3**,**5**,**6*). All patients had a history of ear infections and tympanostomy tube placement, previously associated with NTM disease (*7*). Nine patients had used ototopical medication, including quinolone antimicrobial agents (n = 5), steroids, aminoglycosides (n = 2), or both (n = 2). Clinical signs were nonspecific, with persistent tympanic membrane perforation, chronic painless otorrhea resistant to antimicrobial drug therapy, and hearing loss.

The fact that this disease primarily affects children, with a mean age of 6 years, may be related to age-specific environmental exposures, e.g., playing in sand pits or swimming (*9*). Two patients with *M. abscessus* otomastoiditis are siblings (patients 5 and 6); a clonal relationship between the causative bacteria is possible and should be investigated by molecular typing tools.

Primary isolates were from biopsy material (n = 5) or otorrhea fluid (n = 5) and were positive for acid-fast bacilli by direct microscopy for 9 patients. Five patients had a computed tomography (CT) scan performed, which showed fluid in the mastoid (n = 4), bone erosion of the mastoid (n = 2), and mucosal swelling (all); the Appendix Figure displays typical findings.

The mean interval between first symptoms and diagnosis of *M. abscessus* otomastoiditis was 155 days for (range 14–360 days). Otorrhea unresponsive to antimicrobial drug therapy should raise a clinical suspicion of NTM otomastoiditis (*3*), especially in patients with bone destruction visible on CT images. In patients with otorrhea unresponsive to antimicrobial drug therapy, routine CT scanning and *Mycobacterium* spp. cultures, preferably from tissue biopsies (*1*), may reduce diagnostic delay and prevent further damage.

Patients with *M. abscessus* otomastoiditis received drug treatment for a mean duration of 3 months (range 28–150 days) and 1.8 episodes of surgery. Five patients with *M. abscessus* otomastoiditis received clarithromycin monotherapy; 5 received multidrug therapy with fluoroquinolones (n = 3), fluoroquinolones, rifampin, and ethambutol (n = 1), or meropenem (n = 1 (Appendix Table).

Complications of surgery comprised delayed wound healing (n = 4) and fistula formation (n = 2; Appendix Table). Two patients underwent incus removal and later chain reconstruction surgery (patients 7 and 8). In 1 patient, the infection spread and caused culture-proven cervical lymphadenitis, a retroauricular abscess, fistula, and facial nerve palsy.

Eight patients were eventually cured, defined by symptomatic improvement and in some cases confirmed by negative cultures. Two patients were still receiving treatment at the time of data collection. Five patients had persistent conductive hearing loss after treatment (42%; range 30–80 dB; Appendix Table).

American Thoracic Society guidelines for treatment of soft tissue and bone infections caused by *M. abscessus* advocate 4–6 months of therapy with a macrolide, an aminoglycoside and cefoxitin or a carbapenem, based on in vitro drug susceptibility test results, combined with surgical debridement when possible (*1*). Treatment regimens in this study deviated in duration and content; clarithromycin monotherapy is likely to invoke resistance (*1*) and no evidence supports fluoroquinolone use (*1*). Moreover, use of parenteral agents was limited; its reasoning was not generally captured during file review.

*M. abscessus* otomastoiditis is a serious, potentially emerging condition that affects children who have had previous infections, tympanostomy tubes, and ototopical antimicrobial drug or steroid use in the Netherlands. The diagnostic delay and treatment regimens warrant improvement to prevent deterioration, additional episodes of surgery, acquired drug resistance, and to prevent or limit permanent hearing loss.

## Supplementary Material

Appendix Table Clinical data of patients with otomastoiditis caused by Mycobacterium abscessus*

Appendix Figure Computed tomography images of a patient with Mycobacterium abscessus otomastoiditis. Extensive bone destruction in the right mastoid and associated right-sided mucosal swelling can be seen. A) Bone tissue window setting; B) soft tissue window setting.
