# Supplementary material for: Otomastoiditis Caused by Mycobacterium abscessus, the Netherlands
Source: Emerg Infect Dis. 2010 Jan;16(1):166–8. doi: 10.3201/eid1601.090473 (PMC2874437; doi:10.3201/eid1601.090473)
Supplement: Appendix Table — Clinical data of patients with otomastoiditis caused by Mycobacterium abscessus* [file 09-0473_appT-s1.pdf]

Publisher: CDC; Journal: Emerging Infectious Diseases
Article Type: Letter; Volume: 16; Issue: 1; Year: 2010; Article ID: 09-0473
DOI: 10.3201/eid1601.090473; TOC Head: Letter

Appendix Table. Clinical data of patients with otomastoiditis caused by *Mycobacterium abscessus**

Patient no.	Age, y/sex	Predisposing factors	Side	Symptoms	Cultures/ pos/(AFB)	Days to diagnosis	Treatment (n = mo)	Outcome
1	2/M	TT, OD	Right	O, H, P, F, M	5/2 (-)	147	XW-X-X, AD, 1RE3ECipCla	Cured, H
2	10/F	TT	Right	O, H, P, fi, M	5/1 (+)	330	XW-X-X, 2CipCla	Cured
3	4/M	TT, OD	Left	O, H, P, F, M	2/1 (+)	60	X, 3Cla	Cured, H
4	4/M	TT, OD	Left	O, P	2/1 (+)	14	1Cla-8Cla	Failure
5	5/M	TT, OD	Left	O, H	3/3 (+)	100	4Cla	Cured
6	3/M	TT, OD	Right	O, H, P, M	4/3 (+)	60	X, 5Cla-X	Cured
7	10/F	TT, OD	Right	O, H, P, M, L	1/1 (+)	360	2CipCla-X, CR	Cured
8	12/M	TT, OD	Right	O, H, P, A, fi, M, F, L	12/8 (+)	270	X- XW-RD, 1ClaMox-X, LY-CR	Cured, H
9	6/F	TT, OD	Left	O, H, A, F, M,T,V, L	4/2 (+)	90	X-X-XW1Cip-X, 5ClaMer, AD	Failure, H
10	5/F	TT, OD	Left	O, H, P,M, V, L	7/3 (+)	120	X-1Cla	Cured, H

*TT, ventilation tubes; OD, otic drops; O, chronic otorrhea; H, hearing loss; P, tympanic membrane perforation A, otalgia; F, fever; fi, fistula; M, mastoiditis; F, facial nerve palsy; V, tinnitus; V, vertigo; L, lymphadenitis (culture proven); X, surgery; RD, radical debridement; CR, chain reconstruction; Cla, clarithromycin; Rb, rifabutin; Cip, ciprofloxin; R, rifampicin; E, ethambutol; Mer, meropenem; Mox, moxifloxacin; AD, retro-auricular abscess drainage; LY, cervical lymphnode excision; W, delayed wound healing.
