# Supplementary figures and images for: Otomastoiditis Caused by Mycobacterium abscessus, the Netherlands
Source: Emerg Infect Dis. 2010 Jan;16(1):166–8. doi: 10.3201/eid1601.090473 (PMC2874437; doi:10.3201/eid1601.090473)

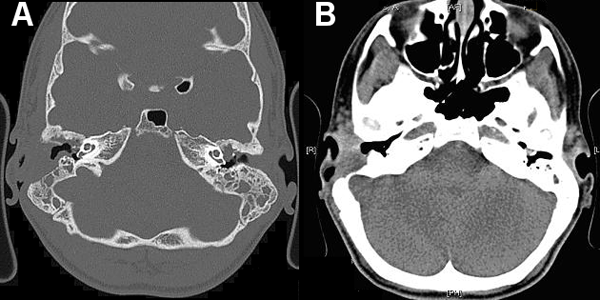

Supplement: Appendix Figure — Computed tomography images of a patient with Mycobacterium abscessus otomastoiditis. Extensive bone destruction in the right mastoid and associated right-sided mucosal swelling can be seen. A) Bone tissue window setting; B) soft tissue window setting. [file 09-0473_appF-s2.gif]
